# Exploring the nature of science through courage and purpose: a case study of Charles Darwin’s way of knowing

**DOI:** 10.1186/s40064-016-3053-0

**Published:** 2016-09-13

**Authors:** Joel I. Cohen

**Affiliations:** 1 Montgomery County Public Schools, Rockville, MD USA; 2 Graduate School USA, Washington, DC USA; 3 Audubon Naturalist Society, Chevy Chase, MD USA

## Abstract

**Introduction:**

In 1836, Charles Darwin returned to England with finches classified and seemingly showing little resemblance. However, subsequent examination by John Gould revealed 13 closely related species endemic to the Galápagos Islands. Despite initial confusion, and Darwin’s overlooking to label these birds by island, some 100 years later they had become evolution’s icon. The same could be said of Darwin’s education and scientific pursuits, beginning in a rough, trial and error manner, lacking direction, but eventually benefitting from an unexpected opportunity that would lead to his theory of natural selection.

**Case description:**

This case study examines Darwin’s way of learning and the reserve of courage and perseverance that he would need to see his treatise on evolution and natural selection published. To do this, themes from studying the “Nature of Science” are used to examine how Darwin’s “way of knowing” advanced before and after his voyage upon *HMS Beagle.* Five themes from the “Nature of Science” were selected to illustrate Darwin’s struggles and triumph: creating scientific knowledge is a human endeavor, such knowledge can explain an order and consistency in natural systems, knowledge comes from a scientist’s way of knowing, is open to revision, and based on empirical evidence.

**Discussion and evaluation:**

The “Nature of Science” as applied to Charles Darwin is explored through the three above mentioned themes identified by the Next Generation Science Standards. Together, the themes help explain Darwin’s way of knowing, from boyhood to manhood. This explanation helps humanize Darwin, allows students to see how he arrived at his theories, how the time taken to do so wore on his health and safety, and the risk Darwin had to weigh from their eventual publication.

**Conclusions:**

Each theme ends with a summary and related extension questions to draw students into the case, and facilitate inquiry. They relate Darwin’s way of learning from the 1800s and his commitment to see his work published, to the learning environment of students today.

## Background

Introducing biography to amplify relevance and meaning of scientific discovery in the classroom has begun (i.e., Williams et al. 2016; Clough 2016). However, there are few such texts that combine a “Nature of Science” approach to the biography and discoveries of a given scientist. This case study examines Charles Darwin’s path towards evolutionary theory by examining key junctures in his life organized through five themes defining the “Nature of Science” (NGSS Lead States 2013; McComas 2015).

In so doing, Darwin’s theories of evolution and natural selection are put on equal footing with the courage, persistence and risk-taking required to bring forth ideas still essential today. Darwin’s education and what became his profession took root as Victorian naturalists expanded understanding of the biological world. These explorers explained the natural world in ways that challenged those based on Divine intervention, heavenly power, and the belief that species were unable to change over time.

Darwin’s life was rich in experience, the harvesting of facts and making unique observations, all providing a foundation for his theories on the origin and diversity of life on earth. This case study enriches understanding of Darwin’s progression towards these theories by sharing moments of greatest fortune alongside his struggles, despair, and misunderstandings. This approach and its historic context help students “develop an understanding of the enterprise of science as a whole—the wondering, investigating, questioning, data collecting and analyzing,” (NGSS Lead States 2013).

These Standards also suggest that one area suitable for this approach is ‘Darwin’s Theory of Biological Evolution and the Modern Synthesis’ (NGSS Lead States 2013). These two recommendations are addressed and presented in a lesson format, ready for teaching, where students explore along with Darwin, rather than gaining facts as if they were preordained and not products of failings, beginnings, and reinvention.

As Darwin changed, so did his explanations and understating, one reverberating off of the other, gaining from errors as well as from opportunities, sometimes making mistakes he regretted terribly. Allowing students to identify with how Darwin matured scientifically opens the possibility that, “such images can, of course, become internalized by students as goals. Discussion of great scientists likewise may potentially establish role models and such histories may thus help recruit more participants to science,” (Allchin 1992).

The themes selected help condense the scholarship of many into discrete sections, allowing teacher and student to rapidly focus on areas of greatest relevance. The “summary and extension’ paragraphs promote student inquiry. They ask students to reflect on the dynamic, interwoven themes reflecting on the nature of science and answering questions rather than merely professing tenets (Clough 2007). Finally, the case study calls a student’s attention to Darwin’s work so that together, “we have discovered something that was not always known,” (Clough 2007), even though these things may be well known at this present date.

New knowledge is then interwoven with the human elements of science (McComas 2015), such that the “dimensions of individual struggle, creativity, and adventure” are incorporated into the way science can be taught (Eldridge 2009).

## Theme 1: Science is a human endeavor

In this section, Darwin’s education is considered in the light of human endeavor, set amidst the social and cultural setting of the day. His learning began as the study of natural history and being a gentleman naturalist returned to favor in British society. Before the 1800s, the study of natural history languished or was abandoned, and sometimes even despised (Baber 1980). With the beginning of the Victorian era, the clamorous pursuit of all things natural allowed and even encouraged Darwin to pursue natural history outside of the classroom.

In fact, Darwin’s growing interest in nature took on greater importance to him than his poor academic performance in traditional classes. If the years Darwin spent preparing to publish his great thesis on evolution of a species are an example of dedicated human endeavor, then the opposite must be said of his early years. Of this time, Darwin’s father once remarked, “You care for nothing but shooting, dogs, and rat-catching and you will be a disgrace to yourself and your family,” (Darwin 1958).

However, Darwin knew he also had positive educational characteristics that he relied on throughout his school years. As he recalled, I “had strong and diversified tastes, much zeal for whatever interested me and a keen pleasure in understanding any complex subject or thing” (Darwin 1958). As an example, his older brother, Erasmus, “worked hard at chemistry, and made a fair laboratory with proper apparatus in the tool-house in the garden, and I was allowed to aid him as a servant in most of his experiments. This was the best part of my education at school, for it showed me practically the meaning of experimental science” (Darwin 1958).

Darwin’s becoming a naturalist allowed him and other scientists-in-the-making to gain far more from experiential than from formal education. In Darwin’s case, his eagerness to learn led to professional connections and experiences that would shape his future. By making the most of his informal education, he gradually transformed from a disinterested student to someone able and willing to circumnavigate the globe.

Other facets of this era influenced Darwin’s endeavors. To match Victorian goals of expansion and empire, a naturalist would become an expected crew member of naval explorations. This past time allowed people from all occupations, commoner to noblemen, to become masters of their own collections. Over time, many of those starting out as raw amateurs turned into professionals, classifying innumerable species of value to the British Empire.

Rather than being mere curiosities, Darwin’s increasing skills in identification and classification of species, and his reputation as a distinguished collector were becoming rapidly essential to the Empire. This may be one reason that even while Darwin was doing more hunting than studying, no one thought it odd that he spent time with a freed slave, John Edmonstone, learning the art of taxidermy while at Edinburgh University. These skills in particular would later be immensely valuable while collecting quadruped animals and birds during his long voyage.

### The courage of conviction

While several qualities pertain to human endeavor, such as persistence, creativity, reason and logic (NGSS 2013), there is an additional quality that pertains to Darwin, and that is courage. While this may not be obvious yet, Darwin recognized, long before his thoughts on evolution were ready, that dangers may arise during and after its publication. Darwin foresaw dangers in many places, including his marriage, which greatly worried him. It was one thing to publish for an audience many of whom he would never meet, it was quite another to think of publishing a manuscript his wife would find difficult to accept because of her devout adherence to religion.

Darwin was convinced of his solution to evolution’s puzzles long before he married his cousin, Emma Wedgwood. The question then was whether or not to tell her, and his father advised Darwin to conceal his feelings, but his anxieties grew, “not because he thought he was wrong about the origin of species, but because he felt sure he was right. He knew what he had to say would be shocking to Emma and others who believed God were the creator of all species,” (Heiligman 2009).

Despite this deep religious rift, Charles and Emma believed unfailingly in the deep-seated goodness of the other. Even as Charles pursued thoughts that could well brand him, and by association, his family as non-believers, they trusted each other. In fact, if Darwin had published his explanations of evolution in an earlier century, he may well have been called a heretic, and subject to the Inquisition. To advance his ideas on evolution it seemed he must wage a private battle between the religious convictions of the day and the implications of his theory. This was a fight he ultimately accepted, one requiring the greatest courage for over 20 years, beginning from his engagement to Emma.

### Summary and extension

Darwin’s early endeavors are seen very much as outgrowths of the naturalism that surrounded him. His ardent pursuit of nature became a mixture of experiential and academic knowledge rooted in the wisdom of the time, but wisdom which it did not take Darwin long to surpass. *Extension*: How did the era of the explorer-naturalist help Darwin’s endeavors to learn, even while doing poorly in his academic classes? Could such an approach, which is, substituting field work experience for the academic classroom, work today as it did for Darwin?

## Theme 2: Scientific knowledge assumes an order and consistency in natural systems

Darwin’s unstructured and haphazard beginning eventually leads to greater understanding of the order and consistency of nature. Two activities are highlighted here that increased his understanding of order through classification. His first encounter was with beetle collecting. As Darwin noted later in life, “It seems therefore, that a taste for collecting beetles is some indication of future success in life!” (Darwin 1958), and this pastime was certainly predictive of Darwin’s successes.

By 1827, Darwin pursued coleopteran collecting as if it were a fulltime profession. This coincided with declining interests in university classes and dropping grades. His fascination with nature, and an aversion to blood in the operating theaters, cast off any chance of becoming a surgeon and following his father into medicine. However, the beetle collection kept growing, thanks to his coleopteran mentor, Cousin William Darwin Fox. Here Charles met a man determined to fill his Cambridge dorm room with beetles, stuffed swans, incubating moth pupae, and a mounted female goosander shot by Darwin, to name but a few.

Soon, Darwin and Fox were parading about the college grounds, sifting through leaves and seeking out that which had yet to be caught in their nets. It was later in life that Darwin recalled that “no pursuit at Cambridge was followed with nearly so much eagerness or gave me so much pleasure as collecting beetles,” (Darwin 1958). To gain further insight from classifying organisms, Darwin enlisted the help of James Stephens who classified insects for the British Museum (Browne 1995). In total, some 390 unique species were listed at one point from Darwin’s insect collections (van Wyhe 2016).

Darwin’s enthusiasm for entomology pushed him to seek out others who could increase his knowledge. This desire led to his meeting John Stevens Henslow, Professor of Botany at Cambridge University. This friendship was one not only of formal tutelage, but also lessons imparted during their private walks through the surroundings of Cambridge. Darwin had much to learn from such an esteemed don, and he impressed Henslow with his eagerness. The professor would ultimately help secure Darwin’s place aboard the HMS *Beagle*.

Henslow introduced Darwin to Adam Sedgwick, Woodwardian Professor of Geology at Cambridge. Henslow persuaded Sedgwick to take Darwin on his annual travels to North Wales where he studied the oldest rock formations. This would be Darwin’s second encounter with order and consistency in nature. Through their travels, Darwin discovered much that prepared him for geologizing in faraway lands. The geology principles he learned from Sedgwick led Darwin to understand the formation of rock and rock layers, and the geologic time required for these changes to occur.

However, Darwin still had much to learn of geological eras and the organisms that belong in each. For example, during their travels, Darwin met a laborer who told him of a sea shell found in a pit being dug. Darwin, in his inexperience, was quite excited and made much of this discovery, believing that the shell belonged there, and had just been exposed by the laborer’s digging. However Sedgwick explained that, “it must have been thrown away by someone into the pit. If really embedded there it would be the greatest misfortune to geology, as it would overthrow all that we know about the deposits,” (Darwin 1958). In less than a week, Sedgwick saw Darwin grow from one who understood how wrong he had been about the shell, to one now proficient at measuring angles of elevation, interpreting rock strata, and able to “generalize from his observations,” (Desmond and Moore 1991).

Whether it was classification of rocks and minerals encountered with Sedgwick or the beetles he named and classified with his cousin, he was becoming a talented observer of nature’s order, and sought explanations for that order. His eagerness never diminished. He realized that nature has an order and that relations within that order exist as much for rocks in formation as for beetles among the coleopteran.

### Summary and extension

Through experiences in collecting and classifying, Darwin came to understand the patterns of nature, and how important they are for establishing relations among species. *Extension*: How did Darwin’s beetle collecting and observations made with Sedgwick help him understand the importance “of order in nature”? How in fact did that occur?

## Theme 3: Science is a way of knowing

Science represents not only a body of knowledge, but processes and practices that enable one to go beyond currently established knowledge. Darwin’s passage aboard the *HMS Beagle* provided the impetus of thought needed to surpass the body of knowledge he had already assimilated. While one reads of Darwin’s discoveries, it is easy to presume that his place aboard the *Beagle* was never in doubt. In fact, given his youthful age and lack of professional experience, is it not more of a surprise that one who had never left the shores of England was entrusted with such a positon? How in fact did such a thing occur?

### Securing passage

Darwin’s place aboard ship was not earned from years of professional study, but rather from good fortune, recommendations from his mentors, and the fact that others had already turned down the invitation. While Darwin’s greatest insights and theories came long after the voyage, it provided a most important opportunity and Darwin was quick to see how such a voyage would expand his way of knowing. The process leading to his selection as ship’s naturalist demonstrates Darwin’s immediate pursuit of such an opportunity, even though there would be numerous obstacles to face in gaining approval. Here was a chance to pursue earlier dreams of exploring the tropical world, but to do so required him pursuing each of many steps needed to secure passage.

The remarkable opportunity to serve aboard ship as a naturalist and companion for the *HMS Beagle’s* Captain, Robert FitzRoy, began with a letter from Steven Henslow informing Darwin of the Admiralty’s interest in securing a qualified individual to serve aboard the *Beagle*. Upon seeking his father’s approval, Doctor Robert Darwin made it clear that he would never allow his son to depart on such a dangerous voyage, especially one offering no future employment.

However, Darwin’s Uncle, Josiah Wedgwood, was willing to speak to Darwin’s father on the matter, and succeeded in convincing Darwin’s father to put aside his reluctance, and grant his son this opportunity. This was no little commitment, for aside from being concerned with his son’s safety, Robert Darwin had to finance his son’s passage for a five-year mission. At that moment, Darwin’s fate had depended upon his Uncle’s willingness to travel to the family home in Shrewsbury and confront this mighty man on behalf of his son. If Uncle Josiah had failed to persuade the Doctor, his son would have never been aboard ship when it sailed.

As Darwin later recorded, “The voyage of the *Beagle* has been by far the most important event in my life, and has determined my whole career; yet it depended on so small a circumstance as my uncle offering to drive me thirty miles to Shrewsbury, which few uncles would have done, and on such a trifle as the shape of my nose.” (Darwin 1958).

Upon gaining his father’s approval, Darwin’s next challenge was to have an interview with Captain FitzRoy and seek his approval. In addition to being an able seaman, FitzRoy was a self-made expert in phrenology, using observations of the skull’s shape to indicate strengths or weakness of different faculties. As their interview ended, the Captain reflected on Darwin’s facial features. He thought they indicated a lack of resolution. The Captain “… doubted whether anyone with my nose could possess sufficient energy and determination for the voyage (Browne 1995).

However, FitzRoy recanted, and finally his place onboard was secured with the support of his father, and the approval of the Admiralty. Darwin’s eagerness and lack of other concerns led to an adventure that others were unable to pursue, and that would change his life and scientific career forever.

### Way of knowing

When the *HMS Beagle* left England in 1831, its primary mission was to chart the South American coastline. Darwin’s appointment as the ship’s naturalist enabled his study and observation of all things natural wherever ship dropped anchor. This included geological formations, coral reefs, living species, fossils, and the people and cultures he encountered. Like other naturalists of his time (Hemming 2015), the social and local knowledge accumulated for each species collected expanded their way of thiking. Summarily, Darwin’s experiences with diverse cultures opened another avenue of learning, with recollections of extreme anger and disgust over slavery, understanding the use of species in local diets, and picking up on observations he missed, but obtained from local citizens.

Darwin brought books aboard ship containing the most recent knowledge of geology, exploration, and observations of the dense, tropical forests he would encounter. These books, along with those of the *Beagle’s* library, helped build a foundation for considering nature’s diversity, his collecting experiences, and as he began to theorize on all that he was finding. As his confidence grew, he found himself able to “refine, elaborate, revise, and extend this knowledge,” (NGSS 2013). Darwin’s observations guided him to new theories which were astounding at the time, and have remained influential to the present day. As such, the voyage led to a way of knowing that surpassed what he could have accomplished if he had remained in England. The following examples illustrate this fact.

### Fossil mammals

Imagine you were cast back in time to a land of giant mammals stretched across the southeastern shores of South America. Then, you were rushed forward to the year of Darwin’s excursions in 1833. Now, all you could find were smaller, diminutive forms of the giants you saw previously. Some of Darwin’s most remarkable discoveries were the fossil remains of those giant mammals. As he would remark, “The most important result of this discovery, is the confirmation of the law that existing animals have a close relation in form with extinct species,” (Darwin 1839).

Using this new understanding, Darwin was able to “refine and revise” the state of knowledge for these towering animals. For example, he could relate the nine feet long fossilized form of a *Toxodon* to the living form of the capybara, the *Megatherium* to the three-toed sloth, and the *Macrauchenia* to the ever present guanaco. From these specimens, Darwin realized that fossils of extinct animals can closely resemble living species within the same geographical region and that modern forms can be derived from those of long ago.

### Fauna of the Galápagos Islands

In the fall of 1835, the *Beagle* sailed to and around the Galápagos Islands. After visiting four islands, Darwin observed that native to each one were tortoises, mockingbirds, and the finches. He established similarities in climate and geologic formation among the islands, and yet, noticed that they supported animals of close relation, but differing substantially in structure and eating habits.

Darwin encountered finch populations on the islands, but most species collected came from two islands: Charles (Floreana) and James (Santiago) (Sulloway 1982a, b). However, while collecting these birds, Darwin neglected to tag each finch by island, perhaps due to his enthusiasm for the moment. While Darwin was well versed in labelling coleopterans, he was still far more familiar with geology. Consequently, most of his time was spent “geologizing” on the Galápagos, not as a well-trained ornithologist. Upon return to the *Beagle*, he did his best to classify the birds, mistakenly identifying them as finches, warblers, American blackbirds, wrens and grosbeaks.

### Summary and extension

Aboard the *Beagle*, Darwin’s opportunities for learning increased dramatically. Two particular examples of how Darwin learned from his experiences were given: unearthing fossils from South America and encounters with animals on the Galápagos Islands. *Extension:* Compare and contrast these two examples of what Darwin learned and the type of knowledge gained that would later to advance his theories evolution.

## Theme 4: Scientific knowledge is open to revision in light of new evidence

A central part of the “nature of science” is that scientific explanations are subject to revision. With new knowledge, came new insights and the need to improve upon old theories. This is a key theme in Darwin’s progression to understanding evolution, as many ideas changed or were updated as a result of his discoveries. Two examples where new evidence and knowledge led to revised interpretations of theory and classification are presented below.

### Coral reef formation

The *Beagle* reached the Keeling Islands in April of 1836. In his *Principles of Geology*, George Lyell adopted a view that coral reefs formed from the tops of submerged volcanoes, allowing the living polyps to grow in the upper reaches only, and rim the creator with coral. However, Darwin had deduced a different view, one relying on subsidence of ocean floor, in conjunction with the elevation of the lands of South America. Such things were in accordance with Lyell’s “compensatory change” in the earth’s crust.

If Darwin’s view was correct, then “coral should be hundreds of meters deep, having grown steadily as its mountain foundation subsided,” (Huxley 2007). Captain FitzRoy arranged for his crew to dredge the bottom of the coral, and indeed, the great expanse of hidden coral was revealed, proving Darwin’s hypothesis to be correct, thus revising the explanation as set forth by Lyell. Darwin was able to show an “inch by inch” approach to the growth of the coral, accumulating great height as the land below subsided (Browne 1995). Thus, Darwin’s studies were able to benefit from, and link, biological evidence and geological structure, laying the basis for modern theories on reef formation and replace Lyell’s earlier theory.

### Re-classifying the Galápagos finches

Upon return to England, Darwin realized that by neglecting the island location from which each bird was collected, he hindered its correct classification. Darwin needed help to clear up this confusion. Fortunately, other members of the crew were willing to provide Darwin with their collections of similar birds, but with each one identified by its respective island. However, finding someone suitable to help with this classification was difficult. He found “… the geologists all willing to render all assistance and exceedingly kind, but the Zoologists seem to think a number of undescribed creatures rather a nuisance,” (Browne 1995).

Darwin was relieved when scientists at the Zoological Society of London presented the birds to John Gould for classification. Eventually, Gould’s painstaking work revealed these “undescribed creatures” from the Galápagos to be 13 species of closely related birds, quite unlike Darwin’s tentative classification as unrelated birds. Their form was so peculiar that Gould placed them in an entirely new group, classified as 13 species, divided among 5 genera.

Within 6 days after receiving them, Gould presented the Galápagos finches as new to science. He deciphered the significance of the graduated differences among beak size. This separated him from other ornithologists of the day by noting that this variability in beak size among the finches was contrary to beak size being one of the most stable family characteristics. Usually, the plumage was highly modified and variable, whereas in the finches, coloration was almost identical (Sulloway 1982a).

In the first edition of Darwin’s *Journal of Researches* (Darwin 1839), in contrast to its second edition (1845, Darwin gave virtually no attention to the finches. While Gould deserves the credit for unravelling the mystery of the finches, Darwin deserved credit for his continued questioning of the significance of the Galápagos animals as examples of a species’ change over time, which he summarized in the second edition of his records aboard the *Beagle* as: “The most curious fact is the perfect gradation in the size of the beaks in the different species of Geospiza, from one as large as that of a hawkfinch to that of a chaffinch…there are no less than six species with insensibly graduated beaks…Seeing this gradation and diversity of structure in one small, intimately related group of birds, one might really fancy that from an original paucity of birds in this archipelago, one species had been taken and modified for different ends,” (Darwin 1845).

### Summary and extension

This theme contains one of the key truths of the nature of science: all data, publications, research, are to be made available for review and revision. The following questions consider this vital point. *Extension*. Why did Gould’s final classification of the Galápagos finches assume much greater importance than Darwin’s classification of the birds while at sea? How do you think Darwin felt after advancing his theory on the formation of coral reefs, which surpassed and replaced the theory of such laid out by Charles Lyell, his close fined and mentor?

## Theme 5: Scientific knowledge is based on empirical evidence derived from experiment and observation rather than theory

During and after the voyage of the *Beagle*, Darwin patiently accumulated evidence for a species’ ability to change. While his theories eventually followed, it was his astounding, almost unlimited, evidence on the ability of species to change that made his book on evolution so compelling. Secondly, he was able to see and portray relations between any two closely aligned species, as well as those separated in time from one another, as products of common descent. This theme examines his most important publication as an example of theory growing from empirical evidence.

### The origin of species

Theories are explanations for observable phenomena, based on a body of evidence developed over time. By the time Darwin was ready to publish, he had provided a theory for evolution working through natural selection. Darwin was not the only person to arrive at such a theory, as working independently and in another corner of the globe, Wallace (1858) produced such a theory as well. It was indeed remarkable that from both men came to a similar if not equivalent idea—that evolution, meaning change over time, occurred through natural selection.

It was this idea, among many others, that Darwin put forward in his book, *On the Origin of Species by Means of Natural Selection, or the Preservation of Favored Races in the Struggle for Life*. Some 25 years in its conception, the book provides a foundation for evolutionary biology. It removes evolution from prior ignorance, by presenting a scientific theory which allows populations to change over generations, and frees them from the bonds of immutability, as professed by the Church of England. In addition, Darwin proclaimed that the diversity of life arose by common descent with modification allowing for changes in species over time.

All of Darwin’s observations and insights, plus reading of increasing population growth and demand for limited resources, brought him to his theory of natural selection. Briefly, this means that individuals better adapted to their environment survive and increase in number through reproduction. It relies on four propositions which again Darwin had seen or studied. First, living organisms reproduce at rates exceeding their survival. Second, members of a species show variation among individuals, variations which are the basis for beneficial adaptations. Third, there will be competition among members of a species for available resources, and fourth, selection will occur, with the most well adapted surviving.

Darwin explained natural selection in the *Origin* (1859) as, “any variation, however slight and from whatever cause proceeding, if it be in any degree profitable to an individual of any species, …, will tend to the preservation of that individual, and will generally be inherited by its offspring.” In concluding this book, he states, “There is grandeur in this view of life, with its several powers, having been originally breathed into a few forms or into one; and that, whilst this planet has gone cycling on according to the fixed law of gravity, from so simple a beginning endless forms most beautiful and most wonderful have been, and are being, evolved,” (Darwin 1859).

#### Summary and extension

Consider each of Darwin’s observations presented in this study. Which most directly explains evolution through the process of natural selection? For example, would you include the fossils from South America, the speciation of the finches from the Galápagos, formation of coral reefs, Darwin’s beetle collection, or the knowledge gained from his expedition with Sedgwick?

## Conclusion

No matter how the nature of science is defined, it is foremost a personal endeavor, each scientist approaching his or her studies in their own way. In this case study, the life, historical times, and experiences of Charles Darwin illustrate one approach to scientific inquiry, using an experiential approach to understanding nature. However, upon his return to England, a systematic approach for his studies and research, mobilizing evidence for what would become the *Origin of Species*, encompassing the theory of natural selection. In fact, this new approach involved a very distinct and personal schedule once he moved to Down House, one that the entire family observed and by which they set their daily activities.

This study categorized Darwin’s approaches to his studies using five of the themes describing the nature of science as identified by the NGSS. This allows students to explore not only Darwin’s theories, but how he arrived at these findings. Three themes selected are meant to convey the personal and uniquely individual side of the nature of science. As such, extensive information was assembled for the first theme, that of human endeavor, including a section on the courage of conviction. A second essential theme was how one gains knowledge about the order and consistency of nature. This was explored through Darwin’s collecting and categorizing and his experiences aboard the *Beagle* used to illustrate ways of knowing.

The study concludes with examples showing how science is revised, and what Darwin learned from participating in this process. Finally, students consider how empirical evidence was gathered throughout Darwin’s career to support claims and theories that were made in the *Origin of Species*. Examples provided for these five themes shows students how Darwin corrected his earlier errors and grew to become a careful, reflective scientist, despite the lack of promise exhibited earlier in school and when beginning university.

Students leave with the impression of Darwin as a struggling but ultimately successful naturalist, at once marked by disappointments and remarkable opportunities. Rather than see Darwin as a man having all the answers, students understand him as a person working tenaciously to provide evidence for each theory he advanced. Finally, his scientific studies led Darwin to believe that, “The love for all living creatures is the most noble attribute of man,” (Darwin 1859).
